# Characterization of the complete chloroplast genome of *Carallia brachiata* (Lour.) Merr. (Rhizophoraceae)

**DOI:** 10.1080/23802359.2023.2238935

**Published:** 2023-08-16

**Authors:** You Zhou, Jiyun She, Chenzhong Jin, Xiongmei Zhu, Fen Xiao, Jian Zhao

**Affiliations:** a Institute of Forestry, Central South University of Forestry and Technology, Changsha, China; b College of Agriculture and Biotechnology, Hunan University of Humanities, Science and Technology, Loudi, China; c Analysis Department, Biomarker Technologies Corporation, Beijing, China

**Keywords:** *Carallia brachiata*, chloroplast genome, rosid phylogenetics

## Abstract

*Carallia brachiata* (Lour.) Merr. (1919) is an important medical resource distributed across subtropical Asia. In this study, the complete chloroplast genome of *C. brachiata* was sequenced, revealing a total length of 162,460 bp, including four regions – a large single copy (89,814 bp), a small single copy (18,804 bp), and a pair of inverted repeats (26,921 bp each). The overall guanine + cytosine content was 35.76%. In total, 130 genes were annotated within the chloroplast genome, comprising 85 protein-coding, 37 tRNA, and 8 rRNA genes. Subsequent phylogenetic analyses revealed that *C. brachiata* is closely related to *Carallia diplopetala*.

## Introduction

**Figure 1.**
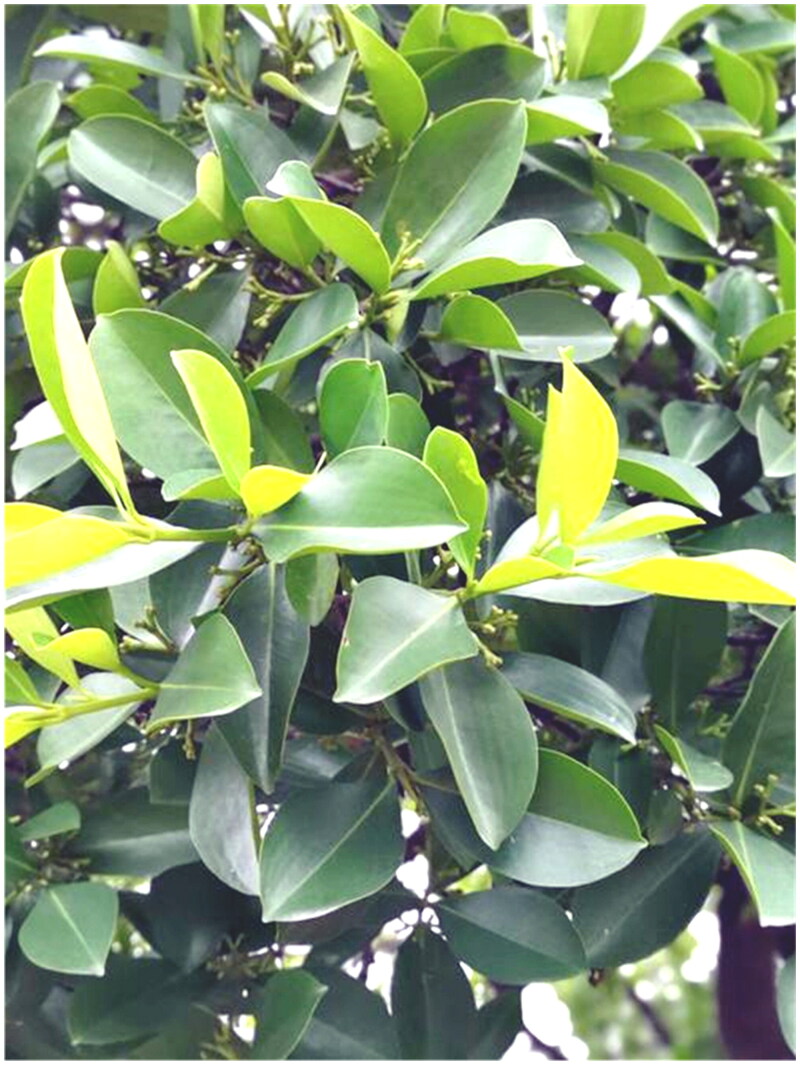
*Carallia brachiata* plant species. The image was taken by the authors in Guangzhou.

*Carallia brachiata* (Lour.) Merr. (1919) is a member of the genus *Carallia* in the family Rhizophoraceae and is mostly distributed across subtropical Asia. Its leaves are oval and have smooth surfaces and edges (Figure 1). It is an important medical resource for treating sapraemia, and its bark is used in pruritis treatment (Ling et al. 2004). However, there is no record of the complete chloroplast genome of *C. brachiata* in the National Center for Biotechnology Information (NCBI) database. Therefore, in this study, the complete chloroplast genome of *C. brachiata* was sequenced, and its phylogenetic position within the Rhizophoraceae was confirmed.

## Materials and methods

**Figure 2.**
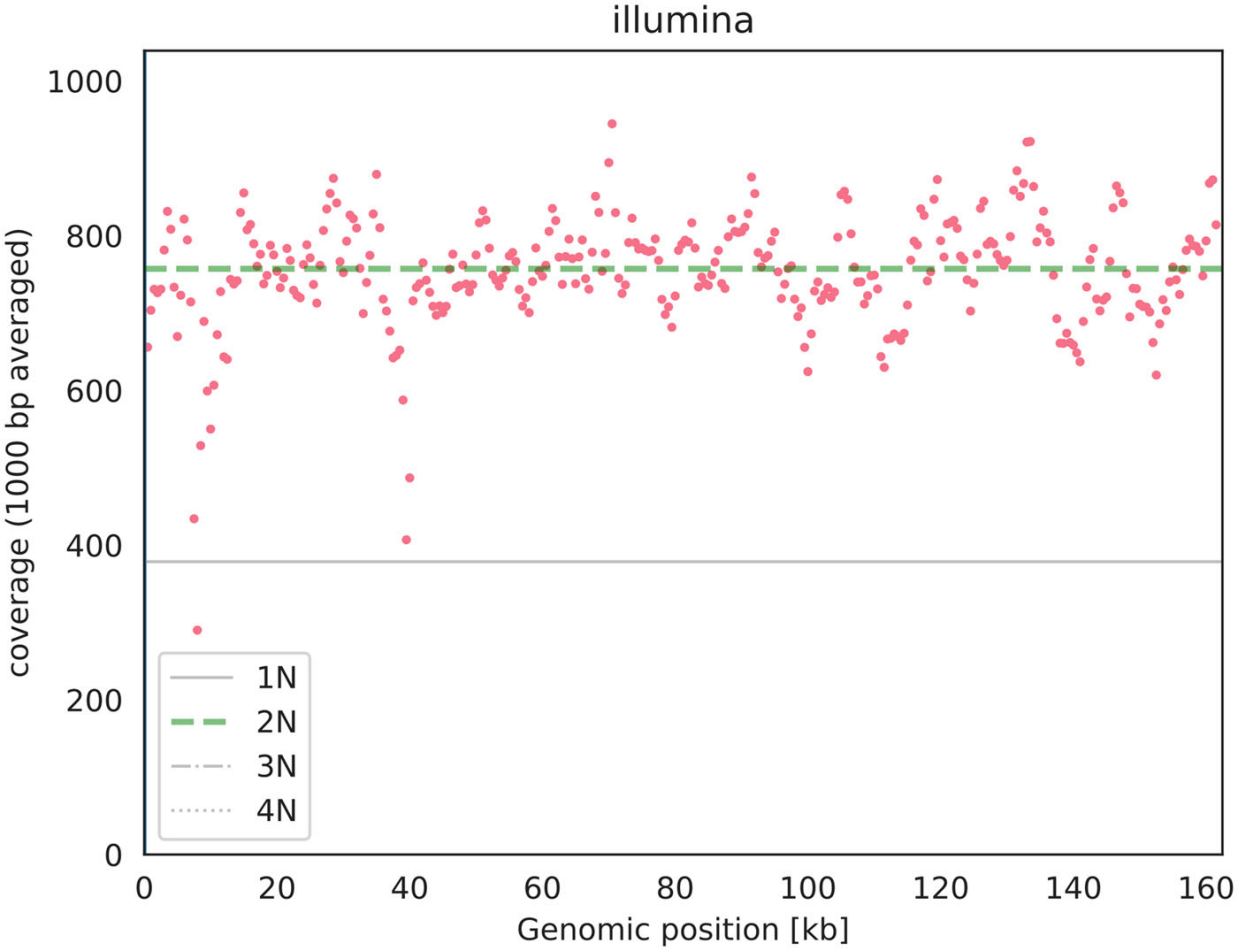
The read coverage depth map of *Carallia brachiata.*

Fresh leaves of *C. brachiata* were collected from the South China Botanical Garden in Guangzhou, China (23°11’16.8" N, 113°22’15.6″ E) in compliance with the national Wild Plant Protective Regulations. The Biomarker Technologies Corporation (Beijing, China) approved the collection of the required samples for analysis. A specimen was deposited at Biomarker Technologies Corporation (Jian Zhao, email: zhaojian0102@outlook.com) under the voucher number ZJS202101110ZJ. The total genomic DNA was extracted from the fresh leaves using the modified CTAB method (Doyle and Doyle 1987), and libraries were prepared using the NexteraXT DNA Library Preparation Kit (Illumina, San Diego, CA). The libraries were then sequenced on the Illumina Novaseq 6000 platform, and the raw data obtained were filtered using PRINSEQlite v. 0.20.4 (Schmieder and Edwards 2011), yielding 3.46 Gb of clean data with a read coverage depth over 600X (Figure 2). High-quality reads were assembled into the chloroplast genome using *de novo* assembler SPAdes v.3.11.0 (Bankevich et al. 2012). Finally, the complete chloroplast genome was annotated using the PGA software package (Qu et al. 2019), with the chloroplast genome of *Pellacalyx yunnanensis* (MN106253) serving as a reference. The results were then submitted to GenBank under accession number OM141003.

Sixty-two homologous protein-coding genes (PCGs) from 26 chloroplast genomes in the NCBI were selected using OrthoFinder v2.3.14 (Emms and Kelly 2015). These were aligned with the *C. brachiata* genome using MUSCLE v.3.8.1551 (Edgar 2004), and conserved sequences were extracted from the alignment using Gblocks v0.91b (Talavera and Castresana 2007). Prottest v3.4 was used to select the HIVb + I + G + F model, and *Couratari macrosperma* (MF359944.1) from Lecythidaceae was used as the outgroup. Finally, IQtree v. 1.6 was used to construct a maximum likelihood tree with 1000× bootstrap resampling (Nguyen et al. 2015).

## Results

**Figure 3.**
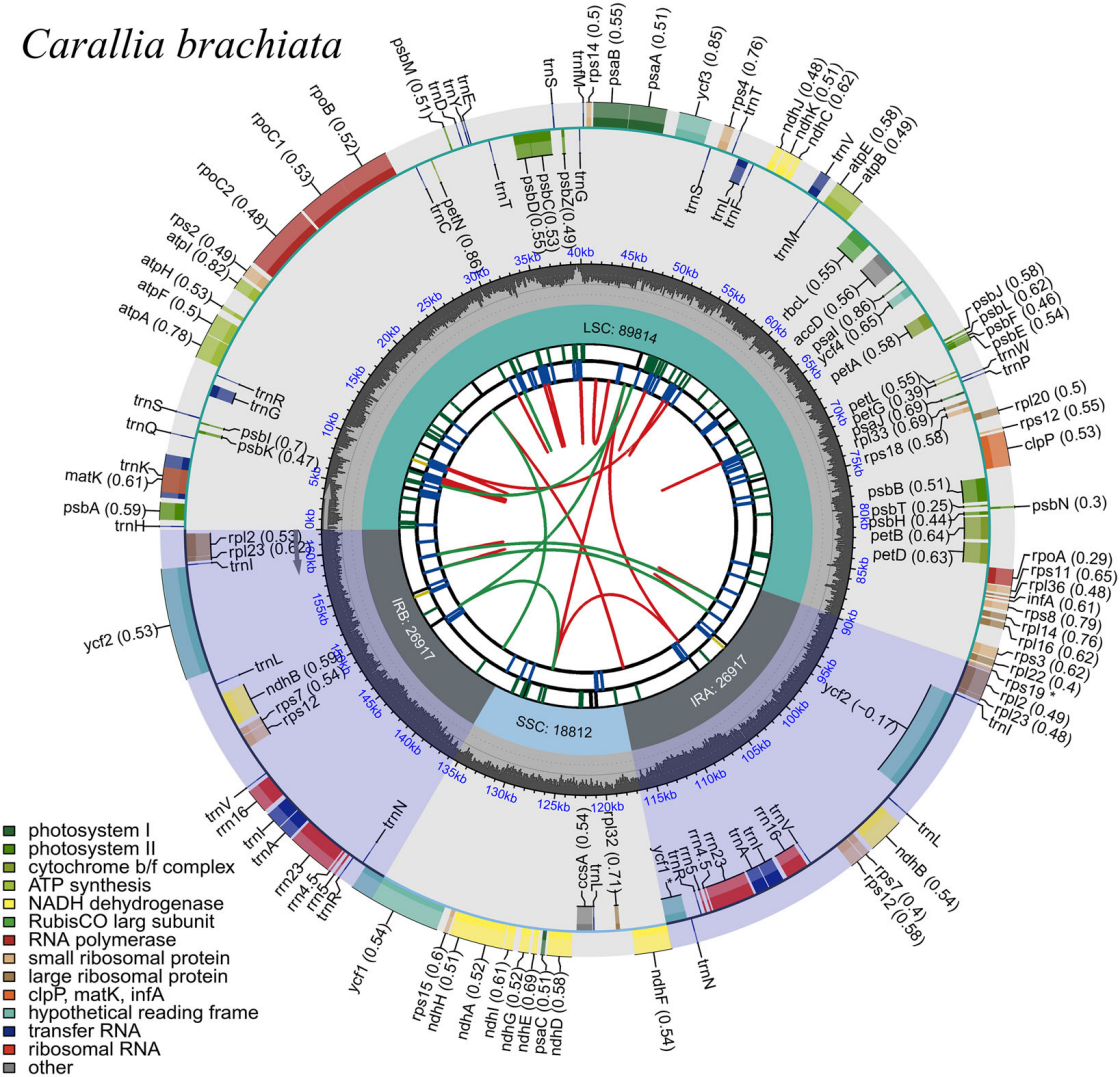
Circular representation of *Carallia brachiata* chloroplast genome, showing the clockwise (genes inside the circle) and counterclockwise (outside) transcribed genes. Colors identify genes from the same functional category, following the figure legends. In the inner circle, the dark and light grey bars indicate the guanine + cytosine and adenine + thymine content, respectively. IRa and IRb: inverted repeat regions; LSC: large single copy region; SSC: small single copy.

The complete chloroplast genome of *C. brachiata* was a typical quadripartite structure that contained 162,460 bp across four areas, including a large single copy (89,814 bp), a small single copy (18,804 bp) and a pair of inverted repeat regions (26,921 bp each) (Figure 3). The total guanine + cytosine (GC) content of the genome was 35.76%. In total, 130 genes were annotated within the chloroplast genome of *C. brachiata*, including 85 PCGs, 37 tRNAs and 8 rRNA genes. Furthermore, 17 genes in the chloroplast genome of *C. brachiata* contained introns. Among them, *trn*K-UUU, *rps*16, *trn*G-UCC, *atp*F, *rpo*C1, *trn*L-UAA, *trn*V-UAC, *pet*B, *pet*D, *rpl*16, *rpl*2, *ndh*B, *trn*I-GAU, *trn*A-UGC and *ndh*A contained a single intron, whereas *ycf*3 and *clp*P had two introns. Owing to their location in the inverted repeat region, 12 genes were duplicated, including one PCG (*ycf*1), four rRNAs (*rrn*4.5, *rrn*5, *rrn*16 and *rrn*23), and seven tRNAs (*trn*I-CAU*, trn*L-CAA, *trn*A-UGC, *trn*I-GAU, *trn*V-GAC, *trn*R-ACG and *trn*N-GUU). Additionally, *rps*12 had three and two exons located on the inverted repeats, indicating that *rps*12 exhibited trans-splicing (supplemental Figure S1). Nine genes including *atp*F, *rpo*C1, *clp*P, p*et*B, *pet*D, *rpl*2, *ndh*B, *ndh*A, *ndh*B, and *rpl*2 are cis-splicing genes (supplemental Figure S2).

**Figure 4.**
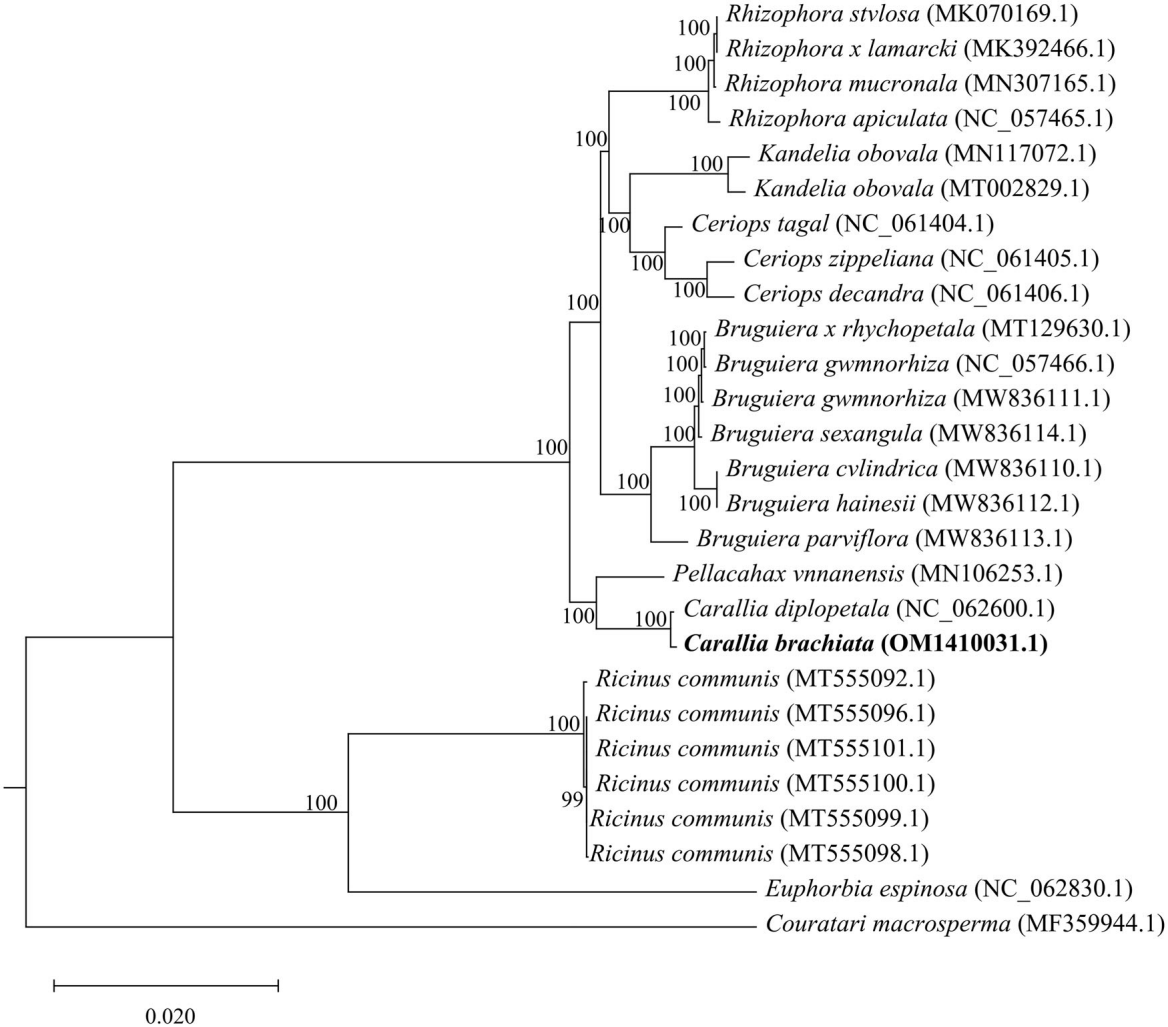
Maximum-likelihood phylogenetic tree for *C. brachiata* and 28 related species based on 62 homologous protein-coding genes. Bootstrap support values are indicated at each node (*N* = 1000). the scale bar indicates the phylogenetic distance in substitutions per site. The following sequences were used: *Kandelia obovata* (MN117072.1) (Du et al. 2019), *Kandelia obovata* (MT002829.1) (Xuli et al. 2020), *Ceriops decandra* (NC_061406.1) (Ruang-Areerate et al. 2022), *Ceriops zippeliana* (NC_061405.1) (Ruang-Areerate et al. 2022), *Ceriops tagal* (NC_061404.1) (Ruang-Areerate et al. 2022), *Rhizophora stylosa* (MK070169.1) (Li et al. 2019), *Rhizophora × lamarcki* (MK392466.1) (Zhang et al. 2019), *Rhizophora mucronata* (MN307165.1) (Wu 2019), *Rhizophora apiculata* (NC_057465.1) (Jiang 2020), *Bruguiera gymnorhiza* (NC_057466.1) (Jiang 2020), *Bruguiera x rhynchopetala* (MT129630.1) (Ying et al. 2020), *Bruguiera gymnorhiza* (MW836111.1) (Ruang-Areerate et al. 2021), *Bruguiera sexangula* (MW836114.1) (Ruang-Areerate et al. 2021), *Bruguiera cylindrica* (MW836110.1) (Ruang-Areerate et al. 2021), *Bruguiera hainesii* (MW836112.1)(Ruang-Areerate et al. 2021), *Bruguiera parviflora* (MW836113.1)(Ruang-Areerate et al. 2021), *Carallia brachiata* (OM141003.1) (this study), *Carallia diplopetala* (NC_062600.1) (Wang et al. 2021), *Pellacalyx yunnanensis* (MN106253.1) (Zhang et al. 2019), *Ricinus communis* (MT555096.1) (Muraguri et al. 2020), *Ricinus communis* (MT555101.1) (Muraguri et al. 2020), *Ricinus communis* (MT555100.1) (Muraguri et al. 2020), *Ricinus communis* (MT555099.1) (Muraguri et al. 2020), *Ricinus communis* (MT555098.1) (Muraguri et al. 2020), *Ricinus communis* (MT555092.1) (Muraguri et al. 2020), *Euphorbia espinosa* (NC_062830.1) (Wei 2021) and *Couratari macrosperma* (MF359944.1) (Vargas et al. 2017).

Twenty-nine species were initially used to construct the phylogenetic tree; however, the bootstrap value was too low to be valid; thus, the related species were removed. As a result, the final phylogenetic tree consisted of 27 species. The phylogenetic analysis revealed that *C. brachiata* was more closely related to *Carallia diplopetala* among all members of the Rhizophoraceae family (Figure 4).

## Discussion and conclusion

In this study, the complete chloroplast genome of *C. brachiata* was sequenced, revealing a total length of 162,460 bp, including four regions: a large single copy (89,814 bp), a small single copy (18,804 bp) and a pair of inverted repeats (26,921 bp each). The overall GC content was 35.76%. In total, 130 genes were annotated within the chloroplast genome, including 85 PCGs and 37 tRNA and 8 rRNA genes. Subsequent phylogenetic analyses revealed that *C. brachiata* is closely related to *Carallia diplopetala* (NC_062600.1). *C. diplopetala*, which exhibits the closest relationship to *C. brachiata,* has a slightly smaller chloroplast genome than *C. brachiata,* comprising 83 PCGs, 37 tRNAs and 8 rRNAs, with a total length of 162,052 bp (Wang et al. 2021).

## Supplementary Material

Supplemental Material

Supplemental Material

Supplemental Material

## Data Availability

The genome sequence data that support the findings of this study are openly available in GenBank (https://www.ncbi.nlm.nih.gov/) under accession no. OM141003. The associated BioProject, SRA, and Bio-Sample numbers are PRJNA801906, SRR17823292, and SAMN25413005, respectively.
