# Supplementary figures and images for: Characterization of the complete chloroplast genome of Carallia brachiata (Lour.) Merr. (Rhizophoraceae)
Source: Mitochondrial DNA B Resour. 2023 Aug 16;8(8):867–71. doi: 10.1080/23802359.2023.2238935 (PMC10435000; doi:10.1080/23802359.2023.2238935)

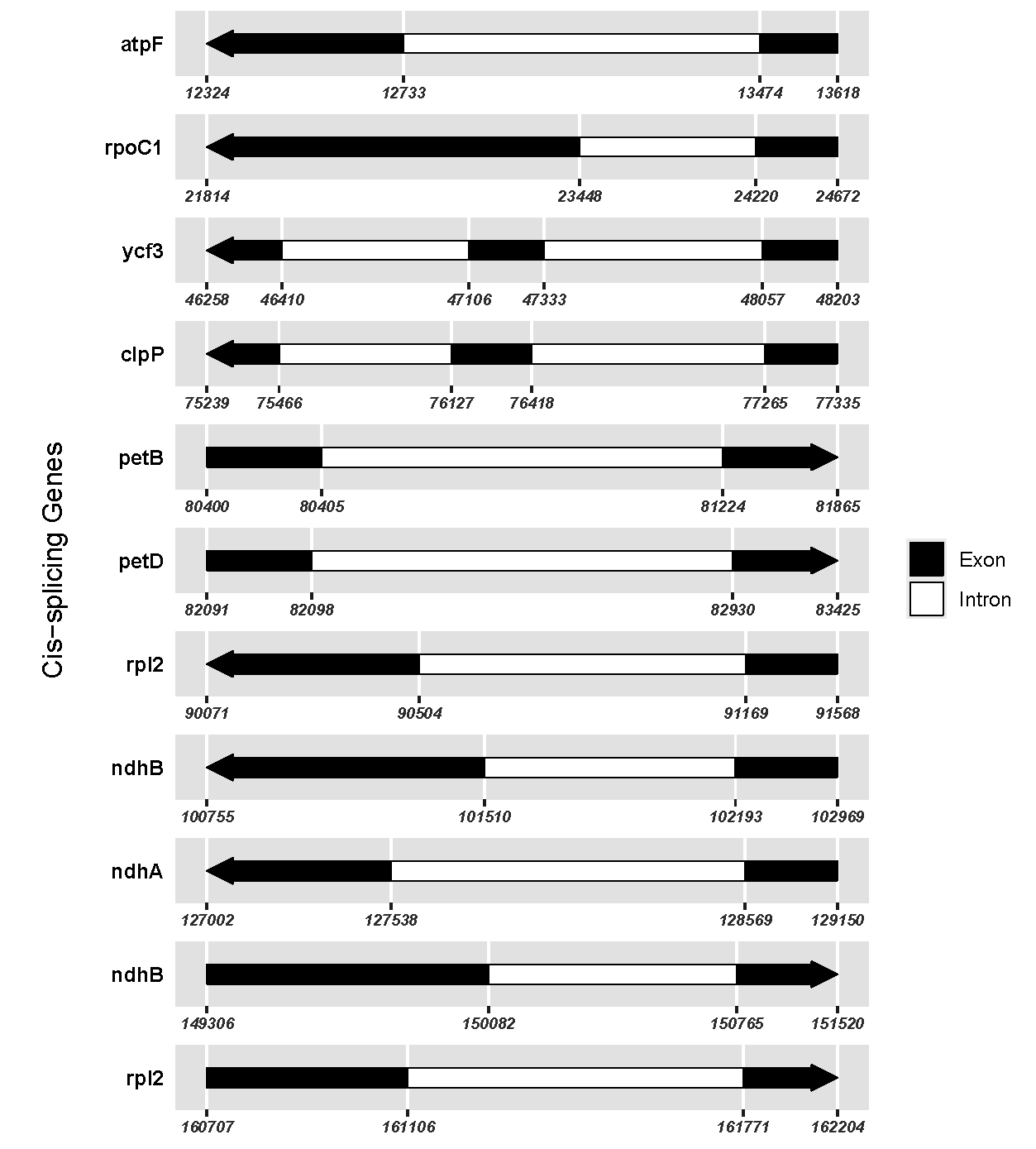

Supplement: Supplemental Material [file TMDN_A_2238935_SM4009.tif]

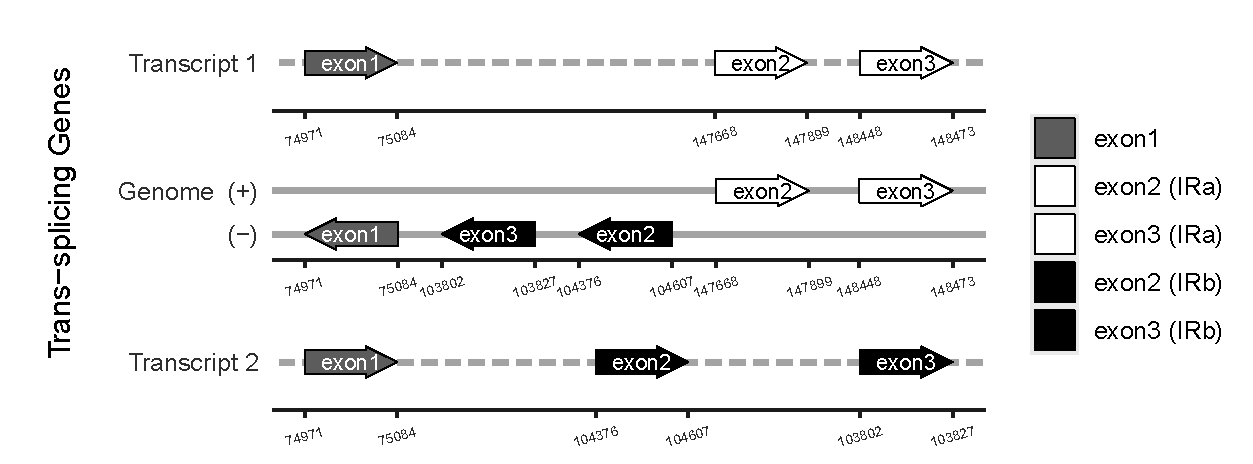

Supplement: Supplemental Material [file TMDN_A_2238935_SM4008.tif]
